# Genetic variants of small airways and interstitial pulmonary disease in children

**DOI:** 10.1038/s41598-021-81280-x

**Published:** 2021-02-01

**Authors:** Mohammed T. Alsamri, Amnah Alabdouli, Alia M. Alkalbani, Durdana Iram, Mohamed I. Tawil, Priya Antony, Ranjit Vijayan, Abdul-Kader Souid

**Affiliations:** 1 Departments of Pediatrics, Tawam Hospital, Al Ain, UAE; 2 Department of Radiology, Sheikh Khalifa Medical City, Abu Dhabi, UAE; 3 Department of Biology, College of Science, United Arab Emirates University, Al Ain, UAE; 4 Department of Pediatrics, College of Medicine and Health Sciences, United Arab Emirates University, Al Ain, UAE

**Keywords:** Clinical genetics, Respiratory tract diseases, Medical genetics

## Abstract

Genetic variants of small airways and interstitial pulmonary disease have not been comprehensively studied. This cluster of respiratory disorders usually manifests from early infancy (‘lung disease in utero’). In this study, 24 variants linked to these entities are described. The variants involved two genes associated with surfactant metabolism dysfunction (*ABCA3* and *CSF2RB*), two with pulmonary fibrosis (*MUC5B* and *SFTP*), one with bronchiectasis (*SCNN1B*), and one with alpha-1-antitrypsin deficiency (*SERPINA1*). A nonsense variant, *MUC5B*:c.16861G > T, p.Glu5621*, was found in homozygous state in two siblings with severe respiratory disease from birth. One of the siblings also had heterozygous *SFTPA1*:c.675C > G, p.Asn225Lys, which resulted in a more severe respiratory disease. The sibling with only the homozygous *MUC5B* variant had lung biopsy, which showed alveolar simplification, interstitial fibrosis, intra-alveolar lipid-laden macrophages, and foci of foreign body giant cell reaction in distal airspaces. Two missense variants, *MUC5B*:c.14936 T > C, p.Ile4979Thr (rs201287218) and *MUC5B*:c.16738G > A, p.Gly5580Arg (rs776709402), were also found in compound heterozygous state in two siblings with severe respiratory disease from birth. Overall, the results emphasize the need for genetic studies for patients with complex respiratory problems. Identifying pathogenic variants, such as those presented here, assists in effective family counseling aimed at genetic prevention. In addition, results of genetic studies improve the clinical care and provide opportunities for participating in clinical trials, such as those involving molecularly-targeted therapies.

## Introduction

Small airways and interstitial pulmonary disease (also known as, childhood hereditary interstitial lung diseases, chILD) refers to complex respiratory disorders characterized by overlapping signs and symptoms of pulmonary dysfunction^1^. These entities often manifest clinically from early infancy^2–4^. A leading cause is surfactant dysfunction, which has been associated with pathogenic variants involving, for example, *ABCA3* (ATP-binding cassette, subfamily A, member 3) and *CSF2RB* (granulocyte–macrophage colony-stimulating factor receptor, beta)^5–9^. Overlapping etiologies include pathogenic variants of *SFTP* (surfactant, pulmonary-associated proteins) family genes and *MUC5B* (MUCIN 5, subtype B, tracheobronchial), which have been associated with idiopathic pulmonary fibrosis^10,11^. Similarly, pathogenic variants of *SCNN1B* (sodium channel, nonvoltage-gated 1, beta subunit) have been linked to a structurally-destructive small airway disease that leads to bronchiectasis^12^. Variants of *SERPINA1* (serpin peptidase inhibitor, clade A, member 1) can cause alpha-1-antitrypsin deficiency^13^.

Currently, the best approach to diagnose these disorders is genetic tests, which lower the cost and need for invasive investigations, such as lung biopsy^14–17^. It is well to know that a reasonable yield of the lung biopsy include findings of fibro-inflammatory changes in autoimmune setting that may warrant specific intervention, and histological analysis in individuals with variants of unknown clinical significance.

The local population of United Arab Emirates include tribes from Arabian Peninsula, Persia, Baluchistan, and East Africa. Founder mutations and autosomal recessive disorders are exceptionally common^18,19^. Many of these diseases may be amenable to prevention through genetic screening and counselling. This study examines the pathogenicity of variations in genes associated with interstitial lung disorders, mainly found in our pediatric patients. Its main purpose is to provide computational and clinical information that improves family counseling. In addition, the results may also improve the clinical care of these children and provide opportunities for participating in clinical trials that involve molecularly-targeted therapies.

## Methods

This retrospective data collection study was approved by ‘Tawam Human Research Ethics Committee’ (SA/AJ/566 on 19th April 2018 and AA/AJ/653 on 19th June 2019). Informed consent to participate in this ‘Retrospective Chart Review’ was exempt. All methods were performed in accordance with the relevant guidelines and regulations.

Our pediatric pulmonary service routinely request genetic studies for children with an unexplained respiratory disease. These investigations are performed by Centogene AG (Germany), and include diagnostic exome sequencing^20^ or Comprehensive Pulmonary Disease Panel (https://www.centogene.com/science/centopedia/comprehensive-pulmonary-disease-panel.html. Accessed 01 June 2020).

Variant information in open databases was combined using Ensembl Variant Effect Predictor^21^. Pathogenicity prediction included scores available in dbNSFP (One-Stop Database of Functional Predictions and Annotations for Human Non-synonymous and Splice Site) for SIFT, PolyPhen, Condel, CADD, FATHMM, LRT, MetaLR, MetaSVM, Mutation Assessor, Mutation Taster, PROVEAN, REVEL, and VEST3^22,23^. Multiple sequence alignment was performed to determine amino acid conservation at sites shown in Table 1, and to compute Jensen-Shannon Divergence (JSD) scores. Amino acid sequences of proteins from *Homo sapiens* (human), *Pan troglodytes* (chimpanzee), *Mus musculus* (house mouse), *Rattus norvegicus* (Norway rat), *Canis lupus familiaris* (dog), *Equus caballus* (horse), *Bos taurus* (bovine), *Xenopus tropicalis* (frog), and *Gallus gallus* (chicken) were collected from NCBI RefSeq and aligned using MUSCLE^24^ in Geneious 9.1.8 (https://www.geneious.com). Aligned sequences were exported in FASTA format to compute JSD. Potential binding pockets in the studied proteins were evaluated using firestar^25^ and 3DLigandSite^26^, post translation modifications were assessed using UniProt^27^, PhosphoSitePlus^28^ and iPTMnet^29^, and functional domains were determined using InterPro^30^. The variants were grouped into three clusters—likely pathogenic, uncertain and likely benign—by k-means clustering in R version 3.6.0, using all pathogenicity scores in Table 1 (Table S1, Supplementary Material). This method yielded *p* < 0.020 on the Kruskal–Wallis test between the three groups for each of the 13 scoring tools. The American College of Medical Genetics (ACMG) classification of the variants from Varsome^31^ was evaluated.

**Table 1:** Studied variants of interstitial lung diseases.

*Variants*	*Phenotypes*	*Frequency*	*JSD*	*SIFT*	*PolyPhen*	*Condel*	*CADD*	*FATHMM*	*LRT*	*MetaLR*	*MetaSVM*	*Mut Assessor*	*Mut Taster*	*PROVEAN*	*REVEL*	*VEST3*	*Varsome ACMG classification*
***ABCA3 (ATP-binding cassette, subfamily A, member 3); SMDP3 (surfactant metabolism dysfunction, pulmonary, 3; MIM#610921); AR***																	
NM_001089.2(*ABCA3*):c.446C > T, p.Ala149Val; missense, rs145483014, MIM#601615, heterozygous	Severe respiratory disease; diffuse patchy ground-glass opacification and interlobular septal thickening on the chest CT at 3 years of age; heterozygous NM_001369.2(*DNAH5*):c.5503C > T, p.Gln1835*	0.000210400	0.76933	0.14 T	0.017 B	0.250 N	22.20	0.93004	0.25994	0.73932	0.76946	0.29422	0.35018	0.33179	0.70615	0.56016	Likely benign
NM_001089.2(*ABCA3*):c.3169G > A, p.Val1057Met; missense, rs537267668, MIM#601615, heterozygous ^**#**^	Infant with pulmonary hypertension; nocturnal hypoxia; upper airway aspiration; recurrent wheezing; mosaic pulmonary attenuation on chest CT	0.000023860	0.71170	0 D	0.195 B	0.463 N	19.23	0.87526	0.27757	0.83545	0.81001	0.62746	0.30797	0.29922	0.65108	0.30296	Uncertain significance
NM_001089.2(*ABCA3*):c.4195G > A, p.Val1399Met; missense, rs763166660, MIM#601615, homozygous	Three siblings with severe respiratory disease (one died at 7 months of age); chest CT images show ground-glass opacification; improved on hydroxychloroquine	0.000007971	0.77892	0 D	0.998 D	0.919 D	25.50	0.94118	0.62918	0.97789	0.98808	0.91273	0.81033	0.60460	0.97887	0.98481	Likely pathogenic *
NM_001089.2(*ABCA3*):c.4675C > T, p.Arg1559*; nonsense, rs769566536, MIM#601615, heterozygous	An individual screened for genetic diseases	0.000008035	–	–	–	–	12.31	–	0.62918	–	–	–	0.81033	–	–	–	Pathogenic
***CSF2RB (granulocyte–macrophage colony-stimulating factor receptor, beta); SMDP5 (surfactant metabolism dysfunction, pulmonary, 5; MIM#614370); AR***																	
NM_000395.2(*CSF2RB*):c.313G > A, p.Val105Ile; missense, rs373460188, MIM#138981, heterozygous	Failure-to-thrive; recurrent wheezing since 2 years of age	0.000286300	0.54853	0.78 T	0 B	0.001 N	0.01	0.62207	0.03557	0.20475	0.10303	0.01225	0.08979	0.03648	0.22963	0.18495	Likely benign
NM_000395.2(*CSF2RB*):c.1381C > T, p.Arg461Cys; missense, rs371045078, MIM#138981, heterozygous	An individual screened for genetic diseases	0.000202900	0.67499	0.01 D	0.792 D	0.703 D	24.60	0.91860	0.18557	0.92149	0.89921	0.48223	0.43436	0.47140	0.86613	0.66657	Uncertain significance
***MUC5B (MUCIN 5, subtype B, tracheobronchial); IPF (pulmonary fibrosis, idiopathic, susceptibility to; MIM#178500); AD***																	
NM_002458.2(*MUC5B*):c.6599G > A, p.Arg2200Gln; missense, rs372137722, MIM#600770, heterozygous	Significant respiratory infections since early infancy; chest radiographs show small airway disease	0.000323800	–	1 T	0.036 B	0.002 N	0.15	0.21114	–	0.12408	0.25032	0.11202	0.08979	0.11931	0.05510	0.17065	Likely benign **
NM_002458.2 (*MUC5B*):c.10352C > T, p.Thr3451Met; missense, rs201116040, MIM#600770, heterozygous	Significant respiratory infections since early infancy; chest radiographs show small airway disease	0.001140000	–	0.12 T	0.007 B	0.261 N	2.24	0.20249	–	0.12760	0.02742	0.14488	0.08979	0.34576	0.16405	0.00785	Likely benign **
NM_002458.2(*MUC5B*):c.14683C > T, p.Pro4895Ser; missense, rs56159668, MIM#600770, heterozygous	Chronic respiratory symptoms limited to severe paranasal sinusitis; extraconal orbital subperiosteal abscess; normal chest radiograph at 5 years of age	0.006217000	–	0.15 T	0.055 B	0.244 N	6.01	0.15731	–	0.07845	0.11250	0.33120	0.08979	0.31762	0.02056	0.12587	Likely benign **
NM_002458.2(*MUC5B*):c.14936 T > C, p.Ile4979Thr; missense, rs201287218, MIM#600770, heterozygous	Two siblings with severe respiratory disease from birth; both required continuous oxygen; one died at 3 years of age	0.000194900	–	0 D	0.261 B	0.472 D	20.00	0.20517	–	0.19680	0.18063	0.54022	0.08979	0.59499	0.22080	0.25021	Likely benign **
NM_002458.2(*MUC5B*):c.16738G > A, p.Gly5580Arg; missense, rs776709402, MIM#600770, heterozygous		0.000024250	–	0.01 D	0.994 D	0.858 D	23.20	0.77474	–	0.86395	0.77567	0.81518	0.22849	0.88119	0.68051	0.46523	Likely benign **
NM_002458.2(*MUC5B*):c.16861G > T, p.Glu5621*; nonsense, MIM#600770, homozygous ^**§**^	Two siblings with severe respiratory disease since birth. ^**§**^	–	–	–	–	–	37.0	–	–	–	–	–	0.81033	–	–	–	Likely pathogenic *
***SCNN1B (sodium channel, nonvoltage-gated 1, beta subunit); BESC1 (bronchiectasis with or without elevated sweat chloride 1; MIM#211400); AD***																	
NM_000336.2(*SCNN1B*):c.616C > T, p.Arg206Trp; missense, rs756809433, MIM#600760, heterozygous	Two cousins with mild bronchiectasis	0.000023860	0.83555	0.12 T	0.111 B	0.269 N	20.30	0.64283	0.17713	0.52702	0.56285	0.43014	0.81033	0.53053	0.36247	0.38410	Uncertain significance ***
NM_000336.2(*SCNN1B*):c.1402G > A, p.Glu468Lys; missense, rs372687977, MIM#600760, heterozygous	Severe respiratory disease since birth; recurrent pneumonia; cricoid cartilage cleft (repaired); recurrent aspiration; lung biopsy showed lipid-laden alveolar macrophages	0.000079540	0.78856	0 D	0.369 B	0.562 D	25.60	0.62940	0.35055	0.56879	0.61414	0.60279	0.43304	0.55295	0.63342	0.79870	Uncertain significance ***
NM_000336.2(*SCNN1B*):c.1871G > A, p.Arg624His; missense, rs549675452, MIM#600760, heterozygous	Chronic sinusitis; two sinus surgeries; failure-to-thrive; heterozygous NM_001369.2(*DNAH5*):c.8765G > A; normal chest radiograph at 10 months of age	0.000048810	0.82138	0.10 T	0.997 D	0.752 D	23.60	0.78655	0.45965	0.72906	0.74423	0.71470	0.41587	0.49314	0.74186	0.44029	Uncertain significance
***SERPINA1 (serpin peptidase inhibitor, clade A, member 1); A1ATD (alpha-1-antitrypsin deficiency; MIM#613490); AR***																	
NM_001002235.2(*SERPINA1*):c.1177C > T, p.Pro393Ser; missense, rs61761869, MIM#107400, heterozygous	An individual screened for genetic diseases	0.000290300	0.79547	0 D	0.985 D	0.877 D	26.20	0.98185	0.23945	0.98873	0.99461	0.92240	0.51338	0.94364	0.94208	0.97397	Uncertain significance
***SFTP (surfactant, pulmonary-associated proteins); IPF (pulmonary fibrosis, idiopathic; MIM#178500; MIM#265120; MIM#610913); AD***																	
NM_001093770.2(*SFTPA1*):c.293G > C, p.Gly98Ala; missense, rs762122985, MIM#178630, heterozygous^**#**^	Infant with pulmonary hypertension; nocturnal hypoxia; upper airway aspiration; recurrent wheezing; mosaic pulmonary attenuation on chest CT	0.000131300	0.70064	0.03 D	0.999 D	0.855 D	23.50	0.99602	0.62918	0.99616	0.96251	0.95208	0.27232	0.75578	0.88551	0.59788	Likely benign **
NM_001093770.2(*SFTPA1*):c.675C > G, p.Asn225Lys; missense, rs150214547, MIM#178630, heterozygous ^**§**^	Severe respiratory disease since birth; frequent intensive care admissions requiring non-invasive ventilation; homozygous *MUC5B*:c.16861G > T	0.000457300	0.86016	0.17 T	0.997 D	0.525 D	17.26	0.62207	0.62918	0.61833	0.67045	0.84584	0.33641	0.88671	0.56865	0.15747	Likely benign
NM_001098668.3(*SFTPA2*):c.73G > A, p.Val25Ile; missense, rs753973926, MIM#178642, heterozygous	Two siblings with atopy (asthma); recurrent sinusitis	0.000019910	–	0.22 T	0 B	0.042 N	0.00	0.13805	0.02909	0.09922	0.28057	0.49323	0.08979	0.13435	0.17396	0.03484	Likely benign
NM_001098668.3(*SFTPA2*):c.572A > G, p.Tyr191Cys; missense, rs866707345, MIM#178642, heterozygous	Chronic wet cough (lost to follow-up); normal chest radiograph at 14 months of age	–	–	0 D	0.934 D	0.825 D	17.52	0.19017	0.14256	0.43612	0.40154	0.87085	0.41461	0.91849	0.39688	0.39330	Likely benign **
NM_000542.3(*SFTPB*):c.1039-6C > G; splice-acceptor, MIM#178640, heterozygous	Respiratory symptoms since birth; improved with age; normal chest radiographs at 2 and 4 months of age	–	–	–	–	–	–	–	–	–	–	–	–	–	–	–	–
NM_003018.4(*SFTPC*):c.176A > G, p.His59Arg; missense, rs201567623, MIM#178620, heterozygous	Chronic wet cough; normal chest radiograph at 5 years of age	0.000224400	0.85794	0.12 T	0.96 D	0.663 D	23.60	0.94392	0.44723	0.95846	0.95521	0.28137	0.81033	0.91164	0.98110	0.66891	Uncertain significance
NM_003018.4(*SFTPC*):c.473C > T, p.Thr158Met; missense, rs1306348581, MIM#178620, heterozygous	Respiratory symptoms since infancy; improved with age	0.000004029	0.61422	0.22 T	0.27 B	0.071 N	2.35	0.79773	0.02210	0.80213	0.64174	0.34626	0.08979	0.45551	0.57130	0.36220	Benign
NM_003019.5(*SFTPD*):c.199 + 9G > A; splice-donor, rs6413522, MIM#178635, heterozygous	An individual screened for genetic diseases	0.00338100	–	–	–	–	5.10	–	–	–	–	–	–	–	–	–	Benign

Allele frequency is from the Genome Aggregation Database (gnomAD). JSD is an amino acid conservation score (higher scores indicate better conservation). SIFT (low scores signify pathogenicity), PolyPhen-2 (high scores signify pathogenicity), Mutation Assessor, Mutation Taster (integrates scores from Ensembl, UniProt, ClinVar, ExAC, 1000 Genomes Project, phyloP, and phastCons), LRT (likelihood ratio test, based on a probabilistic estimation of the phylogenetic relationship, with residue changes treated equally rather than weighting radical or conservative amino acid changes differently), PROVEAN (protein variation effect analyzer), VEST3 (variant effect scoring tool), and FATHMM (*f*unctional *a*nalysis *t*hrough *h*idden *M*arkov *m*odels) are pathogenicity prediction based on alignment of protein sequences and/or structures. Condel (*con*sensus *del*eterious) is a consensus prediction based on SIFT and PolyPhen-2 scores. REVEL (rare exome variant ensemble learner), CADD (*c*ombined *a*nnotation‐*d*ependent *d*epletion; scores range from 1 to 99, e.g., a score of 30 signifies a 0.1% top variant), MetaLR (*meta*-analytic *l*ogistic *r*egression) and MetaSVM (*meta*-analytic *s*upport *v*ector *m*achine) are ensemble-based prediction based on multiple deleteriousness, conservation and ensemble scoring methods derived from surrounding sequences, gene-model interpretations, evolutionary constraints, epigenetic extents, pathogenicity predictions and allele frequencies. B, benign; D, deleterious; T, tolerated; AR, autosomal recessive; AD, autosomal dominant.

*The clinical information is consistent with Varsome ACMG classification.

**The clinical information is inconsistent with Varsome ACMG classification.

***The clinical information suggest pathogenicity.

^**#**^The same patient.

^**§**^One sibling has homozygous *MUC5B*:c.16861G > T plus heterozygous *SFTPA1*:c.675C > G, and one has only homozygous *MUC5B*:c.16861G > T. The one with the two different variants has a more severe respiratory disease (an ‘additive adverse phenotype’). The one with only homozygous *MUC5B*:c.16861G > T had a lung biopsy (Fig. 4).

### Homology modeling

Homology models were generated for variants for which suitable template structures were available. The following Protein Data Bank (PDB) structures were used for modeling: CSF2RB—PDB ID: 2GYS; SCNN1B—PDB ID: 6BQN; and SERPINA1—PDB ID:3NE4. Models were generated using Schrödinger Prime 2019-2 (Prime, Schrödinger, LLC, New York, NY, 2019).

### Statistics

The analyses were performed using SPSS statistical package (version 20). The Kruskal–Wallis H test (non-parametric, k independent samples) test was used to compare groups of variants. The Mann–Whitney U test [nonparametric, 2 independent samples, “Exact Sig (2-tailed)”] was used to compare two groups of variants. *p* < 0.05 was considered significant.

### Ethics approval and consent to participate

This retrospective (Chart Review) data collection study was approved by ‘Tawam Human Research Ethics Committee’ (SA/AJ/566 on 19th April 2018 and AA/AJ/653 on 19th June 2019).

## Results

Table 1 summarizes the pathogenicity of 24 variants of the studied six gene families; two genes are linked to surfactant metabolism dysfunction (*ABCA3* and *CSF2RB*), two to pulmonary fibrosis (*MUC5B* and *SFTP*), one to bronchiectasis (*SCNN1B*), and one to alpha-1-antitrypsin deficiency (*SERPINA1*). Twenty variants are missense, two nonsense, and two intronic. None of the variations in the coding region are at sites known to be post translationally modified based on UniProt^27^, PhosphoSitePlus^28^ and iPTMnet^29^.

**Figure 1:**
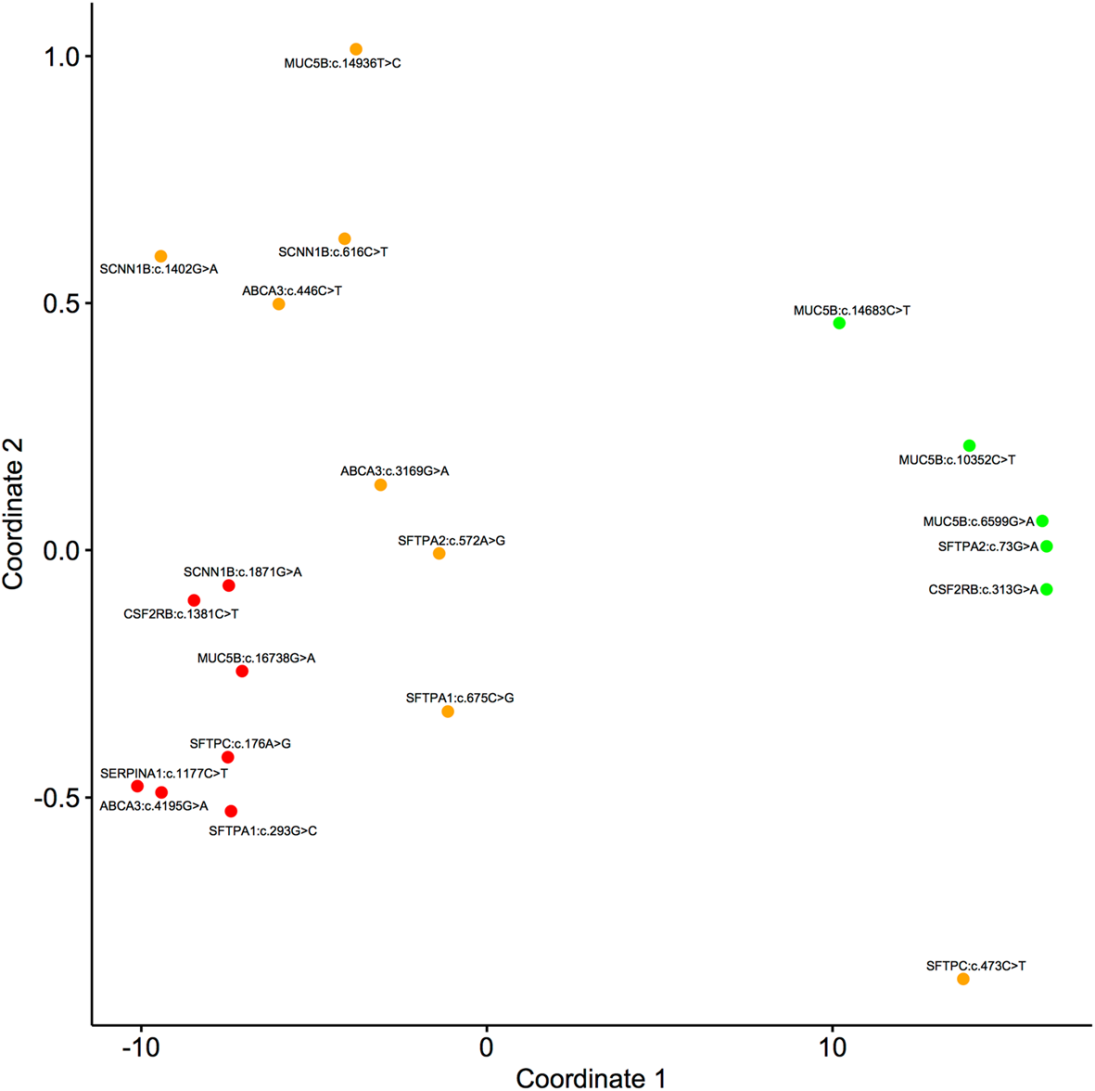
A multidimensional scaling (MDS) plot for the 20 missense variants, using all scores shown in Table 1. The three k-means clusters obtained are colored in red (likely pathogenic), orange (uncertain), and green (likely benign).

Figure 1 shows a multidimensional scaling (MDS) plot for the 20 missense variants, using all pathogenicity prediction scores in Table 1. Seven variants cluster in the lower left zone (‘red’), likely pathogenic with mean ± SD (median) Condel scores of 0.804 ± 0.097 (0.855). Five variants cluster in the middle right zone (‘green’), likely benign with Condel scores of 0.110 ± 0.131 (0.042). The remaining eight variants are in between (‘orange’), ‘uncertain’ with Condel scores of 0.430 ± 0.231 (0.468).

Figure S1 (Supplementary Material) shows ‘dot plots’ for distribution of pathogenicity prediction scores of the MDS plot. The difference between the three clusters is significant for each score (p-values using the Kruskal–Wallis H test): SIFT = 0.0198; PolyPhen = 0.001; Condel = 0.0007; CADD = 0.0009; FATHMM = 0.0024; MetaLR = 0.0005; MetaSVM = 0.00063; Mutation Assessor = 0.01778; Mutation Taster = 0.00725; PROVEAN = 0.00596; LEVEL = 0.00035; and VEST3 = 0.0013.

The difference between the two clusters ‘likely pathogenic’ and ‘likely benign’ for each score (*p* values using the Mann–Whitney U test) is: SIFT = 0.003; PolyPhen = 0.003; Condel = 0.003; CADD = 0.003; FATHMM = 0.003; MetaLR = 0.003; MetaSVM = 0.003; Mutation Assessor = 0.018; Mutation Taster = 0.003; PROVEAN = 0.003; LEVEL = 0.003; and VEST3 = 0.003.

The difference between the two clusters ‘likely pathogenic’ and ‘uncertain’ for each score (*p* values using the Mann–Whitney U test) is: SIFT = 0.675; PolyPhen = 0.006; Condel = 0.002; CADD = 0.009; FATHMM = 0.014; MetaLR = 0.002; MetaSVM = 0.002; Mutation Assessor = 0.232; Mutation Taster = 0.232; PROVEAN = 0.232; LEVEL = 0.001; and VEST3 = 0.014.

The difference between the two clusters ‘uncertain’ and ‘likely benign’ for each score (*p* values using the Mann–Whitney U test) is: SIFT = 0.045; PolyPhen = 0.006; Condel = 0.011; CADD = 0.003; FATHMM = 0.030; MetaLR = 0.003; MetaSVM = 0.006; Mutation Assessor = 0.019; Mutation Taster = 0.030; PROVEAN = 0.011; LEVEL = 0.003; and VEST3 = 0.006.

**Figure 2:**
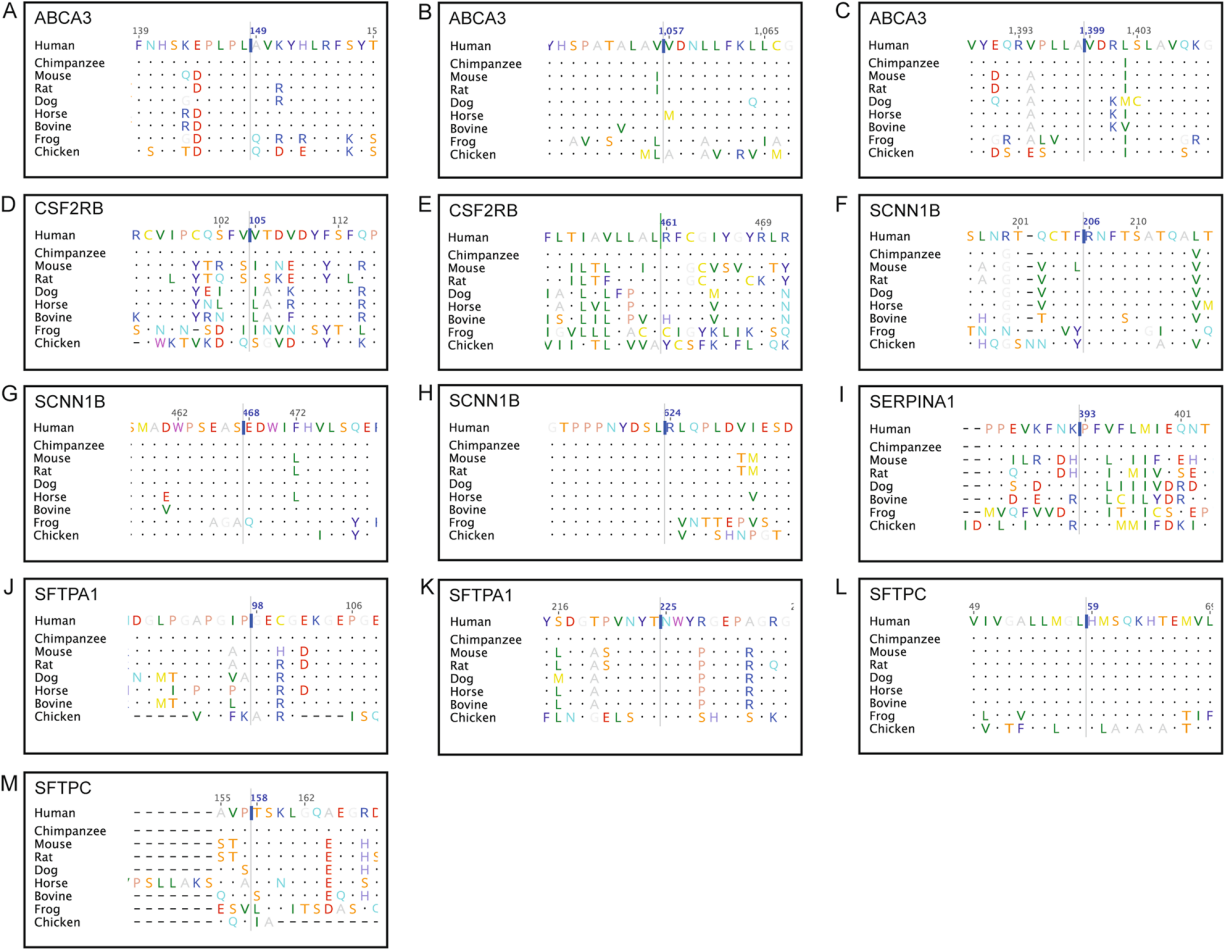
Twenty-one amino acid regions, centered on the missense variation, obtained from a multiple sequence alignment of protein sequences from human, chimpanzee, mouse, rat, dog, horse, bovine, frog, and chicken, where available. (**A**) ABCA3, A1149V (c.446C > T); (**B**) ABCA3, V1057M (c.3169G > A); (**C**) ABCA3, V1399M (c.4195G > A); (**D**) CSF2RB, V105I (c.313G > A); (**E**) CSF2RB, R461C (c.1381C > T); (**F**) SCNN1B, R206W(c.616C > T); (**G**) SCNN1B, E468K (c.1402G > A); (**H**) SCNN1B, R624H (c.1871G > A); (**I**) SERPINA1, P393S (c.1177C > T); (**J**) SFTPA1, G98A (c.293G > C); (**K**) SFTPA1, N225K (c.675C > G); (**L**) SFTPC, H59R (c.176A > G); (**M**) SFTPC, T158M (c.473C > T).

Four autosomal recessive (AR) variants involve *ABCA3*. *ABCA3*:p.Ala149Val has conflicting predictions of pathogenicity (Table 1), mainly due to the high scores of CADD and FATHMM. Ala149 is conserved in mammals (JSD: 0.769, Fig. 2A); it is replaced by Val149, which has a nonpolar sidechain. It is found in heterozygous state with *DNAH5*:p.Gln1835*. Its clinical significance is unknown; Varsome ACMG classification is likely benign. *ABCA3*:p.Val1057Met also has conflicting predictions of pathogenicity (Fig. 1). Val1057 is replaced by methionine in horse (JSD: 0.712, Fig. 2B). The child also has heterozygous *SFTPA1*:p.Gly98Ala. Its clinical significance is unknown; Varsome classifies it as uncertain significance. *ABCA3*:p.Val1399Met has pathogenic scores (Fig. 1). Val1399 is highly conserved (JSD: 0.779, Fig. 2C) and InterPro^30^ indicates that this residue is part of the ATP-binding cassette (ABC) transporter-like domain (IPR003439) of the protein. Evaluation of functionally important residues using firestar^25^ suggests the adjacent residue Ala1398 could be part of the ATP binding site. It is identified in homozygous state in three siblings with severe respiratory disease (one died at 7 months of age). Findings on their chest radiographs and computerized tomography (CT) scans suggest small airway disease (diffuse ground-glass opacification). Both computational and clinical data indicate this variant is pathogenic. *ABCA3*:p.Arg1559* is nonsense (CADD: 12.31). It is found in heterozygous state during screening for genetic diseases. Its clinical significance is unknown; Varsome classifies it pathogenic.

**Figure 3:**
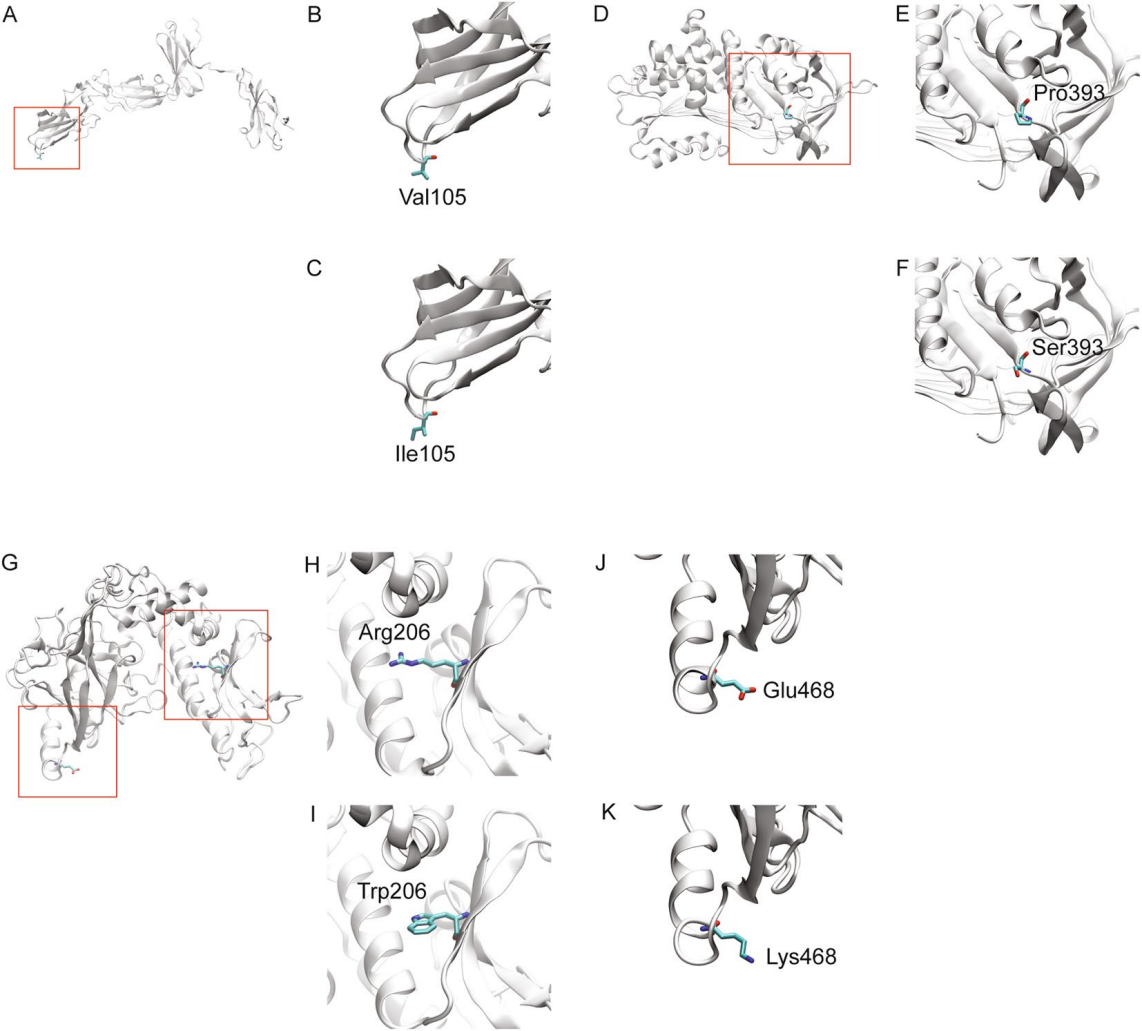
Structural models of wild type and variant proteins. The protein structure is shown in white cartoon representation and the amino acid is shown in stick representation. The red boxed region in each case is enlarged in the subsequent images. (**A**) Structure of CSF2RB with (**B**) wild type Val105 and (**C**) variant Ile105. (**D**) Structure of SERPINA1 with (**E**) wild type Pro393 and (**F**) variant Ser393. (**G**) Structure of SCNN1B with (**H**) wild type Arg206, (**I**) variant Trp206, (**J**) wild type Glu468 and (**K**) variant Lys468.

Two variants involve autosomal recessive *CSF2RB*. *CSF2RB*:p.Val105Ile has benign scores (Table 1). Val105 is not conserved (JSD: 0.548); it is replaced by isoleucine in multiple species (Fig. 2D). In CSF2RB (Fig. 3A), Val105 (Fig. 3B) is located on a solvent-exposed loop. Since Ile105 (Fig. 3C) has similar physiochemical properties, it is not expected to significantly affect the protein structure or function. It is probably benign, in agreement with its Varsome ACMG classification. *CSF2RB*:p.Arg461Cys has conflicting predictions of pathogenicity, mainly due to the low scores of LRT, Mutation Assessor, Mutation Taster, and PROVEAN. Arg461 is conserved (JSD: 0.675); it is replaced by cysteine in frog (Fig. 2E). It is identified in the MDS plot pathogenic (Fig. 1). It is found in heterozygous state during screening for genetic diseases. Its clinical significance is unknown, in agreement with the Varsome ACMG classification of uncertain significance.

Six variants involve autosomal dominant *MUC5B*. *MUC5B*:p.Arg2200Gln, *MUC5B*:p.Thr3451Met, and *MUC5B*:p.Pro4895Ser have consistent benign scores (Table 1). They are identified in children with significant respiratory infections. Here, the clinical information is inconsistent with Varsome ACMG classification of these variants (Table 1). *MUC5B*:p.Ile4979Thr and *MUC5B*:p.Gly5580Arg have conflicting predictions of pathogenicity. Gly5580 is located in the von Willebrand factor type C (VWFC) domain (InterPro ID: IPR001007) of the protein. Both variants are identified in compound heterozygous state in two siblings with severe respiratory disease from birth (one died at 3 years of age). In one sibling, chest radiographs and CT scans at 10 months and 3 years of age show marked perihilar bands of atelectasis and bronchial wall thickening (small airway disease). The clinical information is also inconsistent with Varsome ACMG classification of these variants (Table 1).

**Figure 4:**
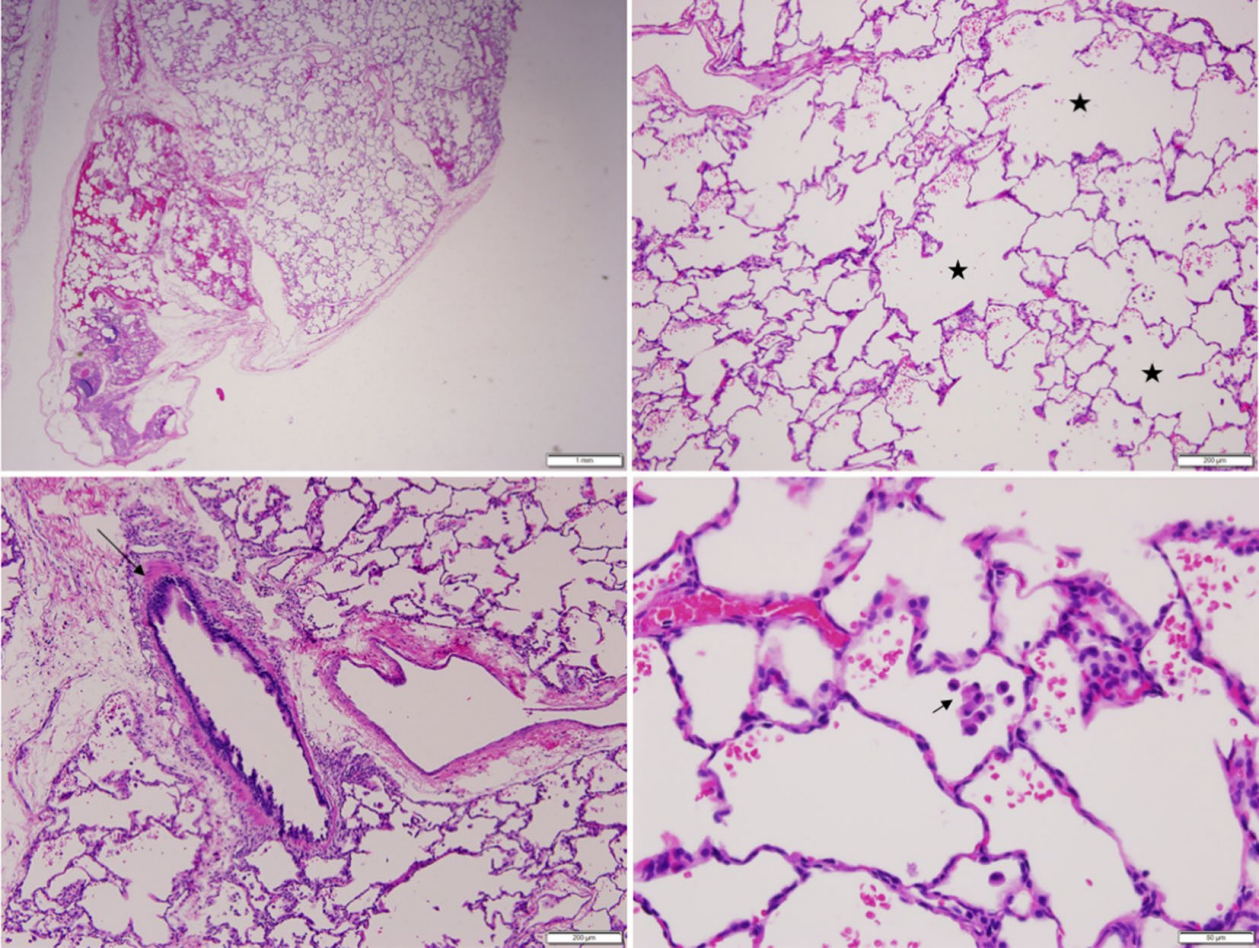
Lung (left lower lobe) biopsy at 18 months of age in the child with homozygous *MUC5B*:c.16861G > T, p.Glu5621*. Hematoxylin and eosin stain showing diffuse enlargement and simplification of the airspaces with thin alveolar septae (stars), mild interstitial fibrosis (long thin arrow), and intra-alveolar macrophages (short thin arrow).

*MUC5B*:c.16861G > T, p.Glu5621* has pathogenic predictions (e.g., CADD: 37.0). It is identified in homozygous state in two siblings with severe respiratory disease since birth. One sibling has homozygous *MUC5B*:c.16861G > T plus heterozygous *SFTPA1*:c.675C > G, and one has only homozygous *MUC5B*:c.16861G > T. The one with the two different variants has more severe respiratory disease (e.g., frequent intensive care admissions). The one with only homozygous *MUC5B*:c.16861G > T had lung biopsy at 18 months of age, which showed significant alveolar growth abnormality (deficient alveolarisation) and interstitial fibrosis (Fig. 4)^32^.

**Figure 5:**
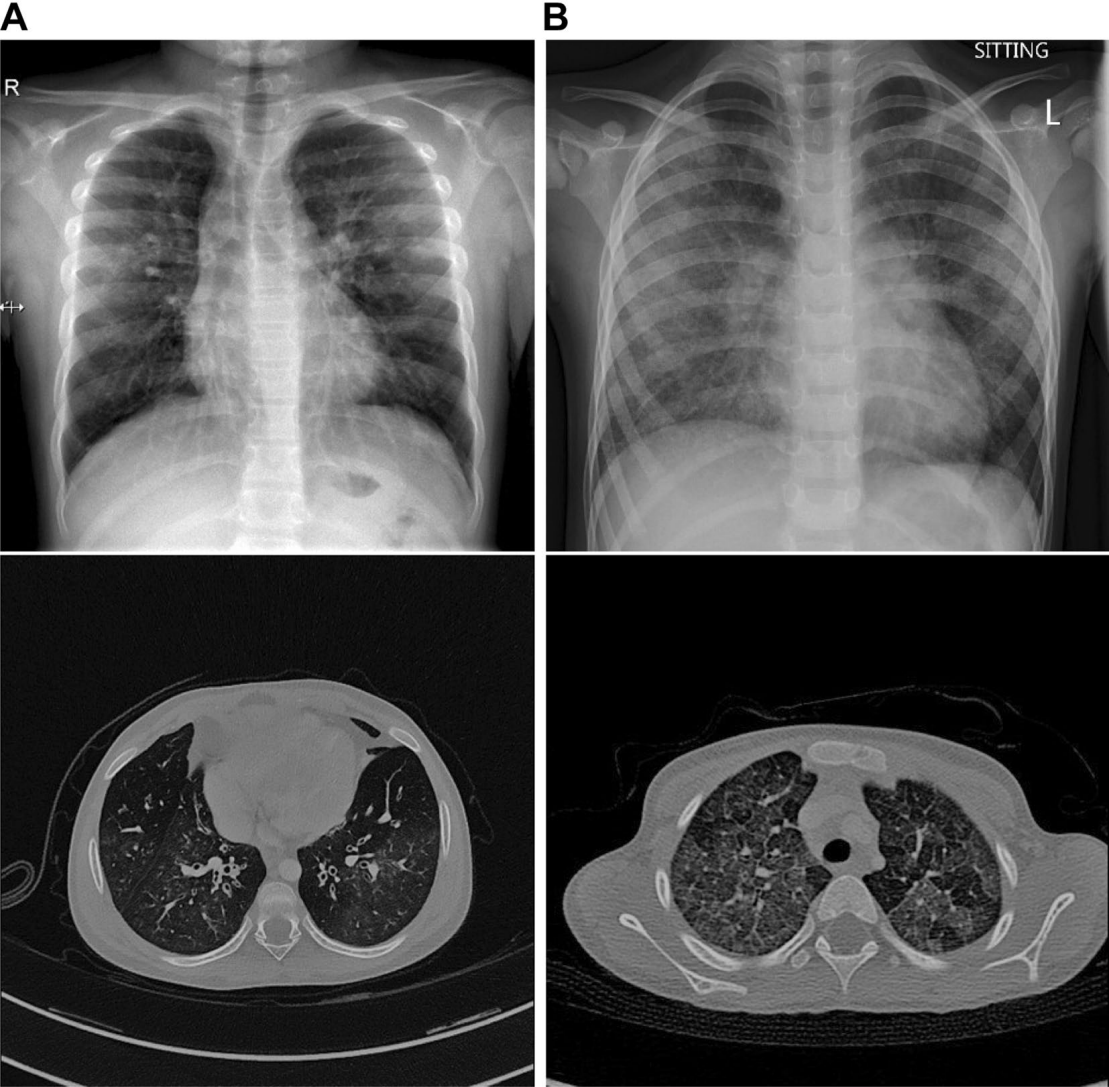
(**A**) Chest radiograph and unenhanced high resolution chest CT axial image of an 18-month-old girl with heterozygous *SCNN1B*:p.Arg206Trp. The chest radiograph shows hyperinflated lungs with bronchial wall thickening and band of atelectasis. The chest CT image demonstrates features of air-trapping, bronchial wall thickening and mild bronchiectasis. (**B**) Chest radiograph and unenhanced high resolution chest CT axial image of an 18-month-old girl with heterozygous *SFTPA1*:p.Gly98Ala. The chest radiograph shows bilateral and quite symmetrical ground-glass opacification, relatively spares the lung apices. The chest CT image demonstrates a combination of septal thickening and alveolar ground-glass opacification creates a typical pattern of crazy-paving.

Three variants involve autosomal dominant *SCNN1B*. *SCNN1B*:p.Arg206Trp has conflicting predictions of pathogenicity (Table 1), mainly due to the high CADD and Mutation Taster scores. Arg206 is highly conserved (JSD: 0.836, Fig. 2F). In SCNN1B (Fig. 3G), Arg206 is located on a solvent exposed β-strand on the surface (Fig. 3H); it does not make notable interactions within the protein. The change to aromatic Trp206 (Fig. 3I), while significant in terms of structure and physiochemical properties, is largely local. This variant is found in two cousins with mild bronchiectasis. Radiologically, the disease mainly involves the small airways (Fig. 5A). The clinical information suggests pathogenicity (Table 1). *SCNN1B*:p.Glu468Lys has conflicting predictions of pathogenicity (Table 1). Glu468 is conserved (JSD: 0.788, Fig. 2G). The negatively charged Glu468 is located on a solvent exposed helix on the surface of the protein (Fig. 3J). The change to positively charged Lys468 (Fig. 3K) is physiochemically drastic. Its location and lack of intramolecular interactions, however, may not affect the protein. It is found in a child with severe respiratory symptoms and cricoid cartilage cleft. His radiological findings are ground-glass opacification and dependent atelectasis; his lung biopsy shows lipid-laden alveolar macrophages. The clinical information suggests pathogenicity (Table 1). *SCNN1B*:p.Arg624His also has conflicting predictions of pathogenicity (Table 1). Arg624 is highly conserved (JSD: 0.821, Fig. 2H). It is found in a child with recurrent sinusitis and normal chest radiograph at 10 months of age. He also has heterozygous *DNAH5*:c.8765G > A. The clinical significance of this variant is unknown.

*SERPINA1*:p.Pro393Ser (autosomal recessive) has consistent pathogenic scores (Table 1). Pro393 is highly conserved (JSD: 0.795, Fig. 2I). In SERPINA1 (Fig. 3D), Pro393 is located at the beginning of a β-strand (Fig. 3E). Physiochemical properties of serine are notably different, and the Ser393 variant (Fig. 3F) is likely to affect the structure or intramolecular interactions in this protein. This variant, also known as Mwürzburg, results in a significant reduction in the level of the enzyme in vitro and in vivo, indicating it could affect the structure and function of the protein^33^. It is found in heterozygous state during screening for genetic diseases.

Eight variants involve *SFTP* (surfactant, pulmonary-associated proteins; autosomal dominant). *SFTPA1*:p.Gly98Ala has pathogenic scores, except for LRT and Mutation Taster (Table 1). Gly98 is conserved (JSD: 0.700), but replaced by alanine in chicken (Fig. 2J). It is identified in a child with severe respiratory disease and a crazy-paving pattern on the chest CT suggesting interstitial lung disease (Fig. 5B). The clinical information is inconsistent with the Varsome ACMG classification of likely benign (Table 1). *SFTPA1*:p.Asn225Lys has conflicting predictions of pathogenicity (Table 1). Asn225, located in the Collectin, C-type lectin-like domain (InterPro ID: IPR033990) of the protein, is highly conserved (JSD: 0.860, Fig. 2K). It is identified in a child with severe lung disease and homozygous *MUC5B*:p.Glu5621*. Its clinical significance is unknown, and its Varsome ACMG classification is likely benign (Table 1). *SFTPA2*:p.Val25Ile has benign scores (Table 1), consistent with the likely permissible replacement of valine with leucine. It is found in two siblings with atopy and recurrent sinusitis. *SFTPA2*:p.Tyr191Cys has conflicting predictions of pathogenicity (Table 1). Tyr191 is located in the Collectin, C-type lectin-like domain (InterPro ID: IPR033990) of the protein. The variant is identified in a toddler with chronic wet cough and normal chest radiograph at 14 months of age; he lost to follow-up. Its clinical significance is unknown, and its Varsome ACMG classification is likely benign (Table 1). *SFTPB*:c.1039-6C > G is found in a toddler with respiratory symptoms since birth, which improved with age. He has normal chest radiographs at 2 and 4 months of age. *SFTPC*:p.His59Arg has conflicting predictions of pathogenicity (Table 1). His59, part of the surfactant protein C, N-terminal propeptide (InterPro ID: IPR015091), is highly conserved (JSD: 0.858, Fig. 2L), favoring PolyPhen (0.96) and CADD (23.6) scores. It is found in a child with chronic wet cough and normal chest radiograph at 5 years of age. Its clinical significance is unknown, and its Varsome ACMG classification is likely benign (Table 1). *SFTPC*:p.Thr158Met has benign scores (Table 1). Thr158, located on the BRICHOS domain (InterproID: IPR007084) of the protein, is not highly conserved (JSD: 0.614, Fig. 2M). The variant is found in a boy with respiratory symptoms since infancy, which improved with age. His chest radiograph at five years of age is normal. Its clinical significance is unknown, and its Varsome ACMG classification is also ‘uncertain significance’ (Table 1). *SFTPD*:c.199 + 9G > A has a CADD of 5.1, and a benign Varsome ACMG classification (Table 1). It is found in heterozygous state during screening for genetic diseases.

## Discussion

The results here show significant respiratory diseases associated with likely pathologic variants, such as *ABCA3*:p.Val1399Met, *MUC5B*:p.Ile4979Thr, *MUC5B*:p.Gly5580Arg, *MUC5B*:p.Glu5621*, *SCNN1B*:p.Arg206Trp, *SCNN1B*:p.Glu468Lys, and *SFTPA1*:p.Gly98Ala. Many of these variants have conflicting predictions of pathogenicity. Therefore, investigating phenotypes associated with such variants is important. Future studies, however, are needed to determine their prevalence in the community. It is worth emphasizing that family (parents and all siblings) genetic studies are important when a pathologic variant is identified. The cost of this endeavor may need to be included in the original agreement between treating institution and investigating laboratory.

Identifying a variant as disease-causing is expected to improve the overall clinical care plan, including counseling. Some of these children may be eligible for lung transplantation, and the variant analysis may be helpful in this regard^17^. Other hopes may include gene therapy and gene editing (when available). Many of these variants are autosomal dominant and, thus, are not directly amenable to prevention by premarital screening. Autosomal dominant disorders, however, are pliable to prevention through a preimplantation genetic testing. This procedure involves in vitro fertilization followed by biopsy of the embryo for genetic testing. The selected embryo is then transferred into the uterus^34^. Thus, a genetic diagnosis is essential for all these serious disorders.

Improved efforts are needed to minimize a delayed diagnosis or treatment. The success with management of cystic fibrosis (including the novel use of specific ATP analogs) should encourage translational research focused on other devastating respiratory diseases, such as chILD. Advancements toward this goal require continual reports on the molecular diagnosis.

Another important information gathered from this study is the conflicting predictions of pathogenicity. For example, *ABCA3*:p.Val1057Met has a SIFT score of zero (damaging) and a PolyPhen score of 0.195 (benign), with a classification of uncertain significance in Varsome and no reports in ClinVar. Another example is *SCNN1B*:p.Glu468Lys with a SIFT score of zero, a PolyPhen score of 0.369 (benign), not reported in ClinVar, and ACMG classification of uncertain significance in Varsome. This autosomal dominant variant is identified in a child with severe respiratory disease. Thus, it is clear that a thorough clinical interpretation of genetic variants is needed. Moreover, clinicians need to provide detailed information on the natural history of the disease for both index case and extended family. In addition, commercial laboratories need to commit to a better investigation of variants, including variants of unknown significance without extra charges.

*MUC5B* has sequences with vastly varying lengths in different species (e.g., human, 5792; chimpanzee, 7982; rat, 4096). This huge gaps potentially affect the pathogenicity scores, as predictors directly or indirectly depend on sequences alignments. Examining *MUC5B* in the dataset is necessary to understand the level of normalization used for this gene. Therefore, the prediction scores for *MUC5B*:p.Arg2200Gln, *MUC5B*:p.Thr3451Met, and *MUC5B*:p.Pro4895Ser may require future confirmation.

In summary, variants associated with interstitial lung and small airway diseases are described here. It is clear that affected children show significant respiratory symptoms at tender age, and the disease advances as time progresses. Genetic tests should also be included in the evaluation of adults with an unexplained lung disease. Homology modeling of the variants may assist in designing compounds that modulate the function of the defective proteins. The results emphasize the use of genetic tests in unexplained respiratory disorders. They also help in generating population based genetic panels for childhood lung diseases.

## Supplementary Information

Supplementary Information

## Data Availability

All data generated and analyzed in this study are included in the article.
