# Supplementary material for: Genetic variants of small airways and interstitial pulmonary disease in children
Source: Sci Rep. 2021 Feb 1;11:2715. doi: 10.1038/s41598-021-81280-x (PMC7851163; doi:10.1038/s41598-021-81280-x)
Supplement: Supplementary file 1 — Supplementary Information [file 41598_2021_81280_MOESM1_ESM.pdf]

Genetic Variants of Small Airways and Interstitial Pulmonary Disease in Children

Mohammed T. Alsamri[1], Amnah Alabdouli[1], Alia M. Alkalbani[1], Durdana Iram[1],

Mohamed I. Tawil[2], Priya Antony[3], Ranjit Vijayan[3,*], Abdul-Kader Souid[4,*]

[1] Departments of Pediatrics, Tawam Hospital, Al Ain, UAE

[2] Department of Radiology, Sheikh Khalifa Medical City, Abu Dhabi, UAE

[3] Department of Biology, College of Science, United Arab Emirates University, Al Ain, UAE

[4] Department of Pediatrics, College of Medicine and Health Sciences, United Arab Emirates University, Al Ain, UAE

malsamri@seha.ae; asmabdouli@seha.ae; alkalbani@seha.ae; diram@seha.ae; mtawil@seha.ae; 201990021@uaeu.ac.ae; ranjit.v@uaeu.ac.ae; asouid@uaeu.ac.ae.

SUPPLEMENTARY MATERIAL

Table S1. Pathogenicity scores used for multidimensional scaling and k-means clustering

Variant	SIFT	PolyPhen	Condel	CADD	FATHMM	MetaLR	MetaSVM	Mut Assessor	MutTaster	PROVEAN	REVEL	VEST3	Cluster
CSF2RB:c.313G>A	0.78	0	0.001	0.013	0.62207	0.20475	0.10303	0.01225	0.08979	0.03648	0.22963	0.18495	Green
MUC5B:c.6599G>A	1	0.036	0.002	0.147	0.21114	0.12408	0.25032	0.11202	0.08979	0.11931	0.0551	0.17065	Green
MUC5B:c.10352C>T	0.12	0.007	0.261	2.242	0.20249	0.1276	0.02742	0.14488	0.08979	0.34576	0.16405	0.00785	Green
MUC5B:c.14683C>T	0.15	0.055	0.244	6.014	0.15731	0.07845	0.1125	0.3312	0.08979	0.31762	0.02056	0.12587	Green
SFTPA2:c.73G>A	0.22	0	0.042	0.001	0.13805	0.09922	0.28057	0.49323	0.08979	0.13435	0.17396	0.03484	Green
ABCA3:c.446C>T	0.14	0.017	0.25	22.2	0.93004	0.73932	0.76946	0.29422	0.35018	0.33179	0.70615	0.56016	Orange
ABCA3:c.3169G>A	0	0.195	0.463	19.23	0.87526	0.83545	0.81001	0.62746	0.30797	0.29922	0.65108	0.30296	Orange
MUC5B:c.14936T>C	0	0.261	0.472	20	0.20517	0.1968	0.18063	0.54022	0.08979	0.59499	0.2208	0.25021	Orange
SCNN1B:c.616C>T	0.12	0.111	0.269	20.3	0.64283	0.52702	0.56285	0.43014	0.81033	0.53053	0.36247	0.3841	Orange
SCNN1B:c.1402G>A	0	0.369	0.562	25.6	0.6294	0.56879	0.61414	0.60279	0.43304	0.55295	0.63342	0.7987	Orange
SFTPA1:c.675C>G	0.17	0.997	0.525	17.26	0.62207	0.61833	0.67045	0.84584	0.33641	0.88671	0.56865	0.15747	Orange
SFTPA2:c.572A>G	0	0.934	0.825	17.52	0.19017	0.43612	0.40154	0.87085	0.41461	0.91849	0.39688	0.3933	Orange
SFTPC:c.473C>T	0.22	0.27	0.071	2.349	0.79773	0.80213	0.64174	0.34626	0.08979	0.45551	0.5713	0.3622	Orange
ABCA3:c.4195G>A	0	0.998	0.919	25.5	0.94118	0.97789	0.98808	0.91273	0.81033	0.6046	0.97887	0.98481	Red
CSF2RB:c.1381C>T	0.01	0.792	0.703	24.6	0.9186	0.92149	0.89921	0.48223	0.43436	0.4714	0.86613	0.66657	Red
MUC5B:c.16738G>A	0.01	0.994	0.858	23.2	0.77474	0.86395	0.77567	0.81518	0.22849	0.88119	0.68051	0.46523	Red
SCNN1B:c.1871G>A	0.1	0.997	0.752	23.6	0.78655	0.72906	0.74423	0.7147	0.41587	0.49314	0.74186	0.44029	Red
SERPINA1:c.1177C>T	0	0.985	0.877	26.2	0.98185	0.98873	0.99461	0.9224	0.51338	0.94364	0.94208	0.97397	Red
SFTPA1:c.293G>C	0.03	0.999	0.855	23.5	0.99602	0.99616	0.96251	0.95208	0.27232	0.75578	0.88551	0.59788	Red
SFTPC:c.176A>G	0.12	0.96	0.663	23.6	0.94392	0.95846	0.95521	0.28137	0.81033	0.91164	0.9811	0.66891	Red

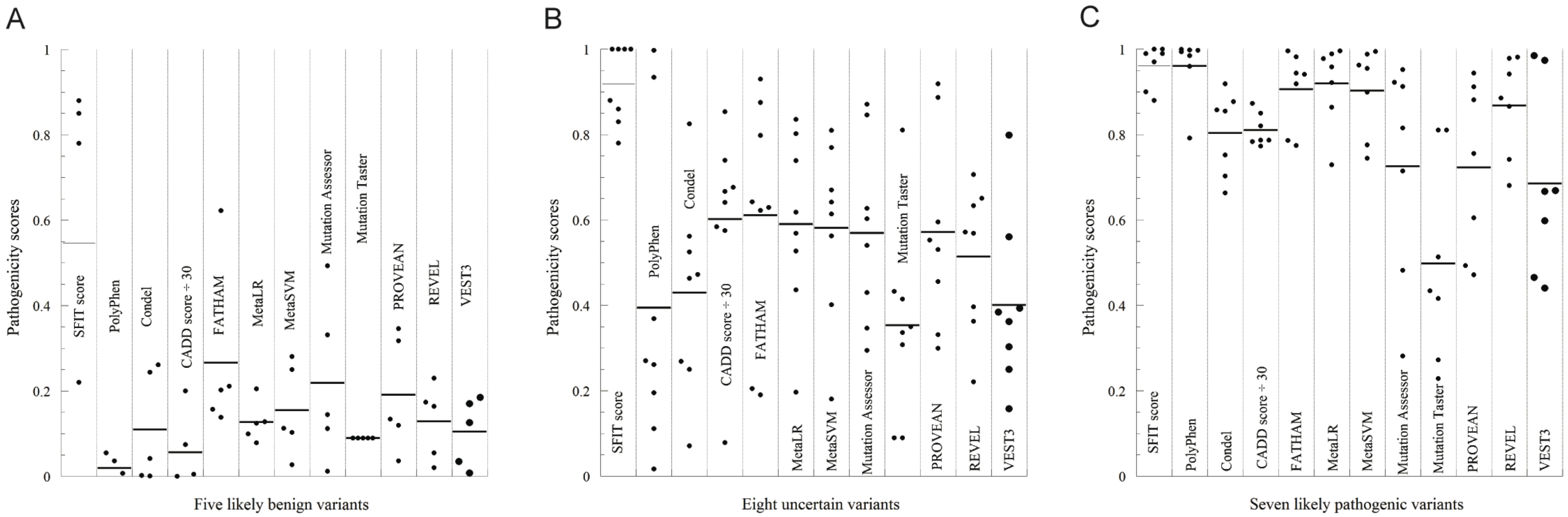

Figure S1. Dot plots showing the distribution of pathogenicity prediction scores for the three clusters – A) Likely benign (Green); B) Uncertain (Orange); C) Likely pathogenic (Red) – of the MDS plot. Horizontal lines are mean.
